# Prospective relationship between family screen time rules, obesogenic behaviours, and childhood obesity

**DOI:** 10.1093/eurpub/ckae169

**Published:** 2024-11-18

**Authors:** Ladan Hashemi, Maryam Ghasemi, Deborah Schlichting, Maryam Pirouzi, Cameron Grant, Boyd Swinburn

**Affiliations:** Violence and Society Centre, School of Policy and Global Affairs, City St George’s, University of London, London, United Kingdom; School of Population Health, Faculty of Medical and Health Sciences, University of Auckland, Auckland, New Zealand; Faculty of Education and Social Work, University of Auckland, Auckland, New Zealand; Department of Paediatrics: Child and Youth Health, School of Medicine, Faculty of Medical and Health Sciences, University of Auckland, Auckland, New Zealand; School of Population Health, Faculty of Medical and Health Sciences, University of Auckland, Auckland, New Zealand; Department of Paediatrics: Child and Youth Health, School of Medicine, Faculty of Medical and Health Sciences, University of Auckland, Auckland, New Zealand; School of Population Health, Faculty of Medical and Health Sciences, University of Auckland, Auckland, New Zealand

## Abstract

Family screen use rules (FSRs) could plausibly protect against the development of childhood obesity, although the mechanisms underlying these protective effects remain largely unexplored. This research aimed to investigate prospectively the associations between exposure to FSRs at age 24 months, obesogenic behaviours (excessive screen time and short sleep duration) at age 45 months, and obesity at age 54 months. Additionally, a model proposing the mediating role of obesogenic behaviours in the association between FSRs and childhood obesity was tested. Data were obtained from 5733 children and their mothers participating in the ‘Growing Up in New Zealand’ study. Logistic regressions examined the association between three FSRs (rules on quality, quantity and timing of screen time, and different numbers of FSRs), obesogenic behaviours, and childhood obesity. Structural equation modelling (SEM) was applied to assess the potential mediating roles of obesogenic behaviours in the association between FSRs and zBMI. Neither exposure to individual nor all three FSRs was significantly associated with lower odds of obesity. However, protective effects of FSRs were observed concerning obesogenic behaviours. Exposure to individual or all three FSRs correlated with reduced odds of not meeting screen time and sleep duration recommendations. SEM analysis indicated no direct association between FSRs and zBMI; nevertheless, a significant indirect association was identified through the mediation of obesogenic behaviours. These findings suggest the potential benefits of promoting the adoption of FSRs as a promising population-based strategy to enhance child health behaviours and mitigate the risk of childhood obesity.

## Introduction 

The rise in excessive screen time among toddlers, around age 2 years, is a growing concern [1] primarily due to the enduring nature of screen time habits established in early childhood [2]. Additionally, early exposure to excessive screen media is associated with adverse health and behavioural outcomes, including shortened attention spans, emotional problems, language difficulties, disrupted sleep patterns, and increased risk of obesity [3–6].

The link between excessive screen time and childhood obesity risk has particularly gained attraction given the alarming rise in obesity rates worldwide. Parents play a pivotal role in shaping children’s screen usage habits by establishing social environments, notably through the introduction and implementation of household screen use rules [7, 8].

Some studies support the protective effects of family screen use rules (FSRs) on screen viewing behaviour. FSRs are defined as the guidelines established by parents to manage their children’s use of digital devices, typically covering aspects such as quantity, quality, and timing of screen use [9, 10]. Research shows that children in households with TV viewing rules have significantly reduced TV viewing time compared to those without such rules [10–12]. However, most studies are cross-sectional and either lack measures of the child’s weight status or present inconclusive findings regarding the link between FSRs and childhood obesity [12, 13].

Longitudinal studies are crucial for understanding how exposure to FSRs in early childhood could influence obesity risk in middle childhood [6, 14], guiding the development of effective strategies to manage children’s screen time and combat childhood obesity.

Moreover, investigating indirect associations rather than direct ones may provide deeper insights into the impact of FSRs during early childhood on later weight status. Although this relationship has not yet been directly studied, we hypothesize that FSRs in early childhood may reduce the risk of obesity in middle childhood by influencing obesogenic behaviours, specifically by reducing excessive screen time and improving sleep duration (see proposed model in Supplementary Fig. S1). Existing research linking FSRs to reduced screen time [1, 14] and its negative association with obesity [12, 14–16] supports our hypothesis. Although the effect of FSRs on sleep behaviours largely remains unexplored, evidence linking excessive screen time to reduced sleep duration [17, 18], and its association with childhood obesity [15, 17, 19] provides additional support for our proposed model.

This study has three main objectives: (i) to investigate prospectively the direct association between FSRs at early childhood (24 months) and childhood obesity outcomes at middle childhood (54 months); (ii) to examine associations between FSRs at early childhood and obesogenic behaviours (i.e. excessive screen time and short sleep duration) at age 45 months; (iii) to explore the pathways through which FSRs at early childhood may reduce the risk of childhood obesity through mediating roles of screen and sleep behaviours.

## Methods

### Participants

Data were obtained from the Growing Up in New Zealand (GUiNZ) study, the largest contemporary longitudinal cohort study of children and their families in New Zealand (NZ). The study recruited 6822 pregnant women with due dates between April 2009 and March 2010 from the Auckland, Counties Manukau, and Waikato District health board regions. The study’s design and recruitment procedures have been described in detail elsewhere [20–22]. The cohort’s characteristics at birth generally aligned with those of the national birth cohort in NZ from 2007 to 2010 [21]. Ethical approval was obtained from the NZ Ministry of Health Northern Y Regional Ethics Committee (NTY/08106/055) and all participating women provided written informed consent. This study utilizes data collected across five waves between 2009 and 2014, following the same children throughout this period. To avoid dependent observations, only one child per family was included, resulting in a final sample size of 5733 children and their mothers.

### Measures

#### Obesity and zBMI

Weight and height measurements were taken when the children reached 54 months, following a standardized protocol that included removing shoes, hats, jackets, or jumpers, and taking duplicate measurements. These values were standardized by age and gender according to the World Health Organization (WHO)’s 2006 Growth Curves. Obesity was defined as a body mass index (BMI) over 30 kg/m^2^ [23] and a binary variable (obese vs. non-obese) was used in binary logistic regression analyses. Additionally, a continuous variable (zBMI) was used in structural equation modelling (SEM). All other information for these preschool-aged children was obtained from maternal responses to closed-ended questions during computer-assisted personal interviews administered by trained interviewers.

#### FSRs

At the 24-month age time point, mothers were asked about FSRs restricting their child’s screen-based behaviours. Three questions considered rules on quality (‘What TV programs your child can watch?’), rules on quantity (‘How many hours of TV, videos, and DVDs can your child watch?’), and rules on timing (‘When your child can watch TV?’). Responses were recorded as ‘Yes’ or ‘No’. If a rule was present, mothers were further asked about the frequency of enforcement. Responses were collapsed into two categories: ‘having the rule’ if enforced most or all the time, and ‘lack of rule’ for other responses (‘half the time’ or ‘never’). A variable indicating the number of FSRs a child was exposed to (ranging from 0 to 3) was then created for analysis.

#### Child’ screen time

At 45 months, screen time was assessed through a parent-proxy report. Mothers reported on their child’s average time spent at home watching TV programming (including free-to-air, online, and pay-TV, or DVDs either on TV or other media) and using electronic media (such as a computer or laptop, including children’s computer systems such as LeapFrog^®^, iPads, tablets, smartphones, and any electronic gaming devices) on a usual weekday. Total daily screen time was calculated by combining TV viewing and electronic media use. A continuous variable was used in SEM analysis. Following guidelines from the NZ Ministry of Health and the WHO for screen viewing among children aged 2–5 years [24, 25], excessive screen time was defined as 1 hour or more per day. A binary variable (excessive screen time: Yes/No) was used in binary logistic regression analyses.

#### Child’s night sleep duration

At the age of 45 months, mothers reported children’s average nightly sleep duration. A continuous variable was used in SEM analysis. Following guidelines from the NZ Ministry of Health and US National Sleep Foundation for preschoolers aged 3–4 years [24, 26, 27], short sleep duration was defined as sleeping <10 hours per night on average. A binary variable (short sleep duration: Yes/No) was used in binary logistic regression analyses.

### Covariates

Covariates included in multivariable analyses were child’s gender, age, and self-prioritized ethnicity reported at age 54 months (NZ European/NZers, Māori, Pacific, Asian, MELAA [Middle Eastern, Latin American or African]), maternal prenatal education level (no secondary school, secondary school, diploma/trade, bachelor qualification, and higher qualification), food security, and neighbourhood deprivation level. The selection of these covariates was informed by literature highlighting the association between sociodemographic factors and childhood obesity [28–30].

The food security index was previously developed using data from GUiNZ [31]. The index scores, ranging from 0 to 49, compromise indicators of poor-quality weaning diets and measures of food poverty, such as the inability to afford food and reliance on food banks. For multivariable logistic regression analyses, the food security score was categorized into two groups: food insecure (below the mean score of 26) and food secure (≥26) [31].

NZ neighbourhood deprivation is reported in the 10-point deprivation index (1 is least deprived). NZDep2006 index at age 2-year interview was used to assess neighbourhood area deprivation [32]. In this study, scores were categorized into three groups: low deprivation (1–3), medium deprivation (4–7), and high deprivation areas (8–10).

### Data analysis

Descriptive statistics on the prevalence of obesity, obesogenic behaviours (excessive screen time and short sleep duration), and exposure to each and all three FSRs for the whole sample and by sociodemographic factors are presented in Table 1. Chi-square tests were used to (1) examine whether obesity, obesogenic behaviours, and FSRs were associated with each of these sociodemographic variables (Table 1) and (2) determine whether obesity and obesogenic behaviours were associated with exposure to each and different numbers of FSRs (0–3) (Table 2).

**Table 1.** Prevalence of exposure to family screen rules (FSRs) at age 24 months, obesogenic behaviours (age 45 months), and obesity (age 54 months) in overall sample and by sociodemographic characteristics

	% (*n*)	Exposure to FSRs			All 3 FSRs %	Obesogenic behaviours		Obesity
		Rule on quality % (*n*)	Rule on quantity % (*n*)	Rule on timing % (*n*)		Excessive screen time (≥1 h/day) % (*n*)	Short sleep duration (<10 h/night) % (*n*)	WHO definition % (*n*)
**Overall sample % (*n*)**	5733	69.4 (3541)	57.0 (2919)	58.9 (3025)	43.7 (2504)	69.2 (3855)	33.6 (1873)	4.5 (255)
**Child’s gender**		%	%	%	%	%	%	%
Boys	51.4 (2945)	69.6	57.3	58.1	43.8	69.7	32.8	5.2
Girls	48.6 (2788)	69.3	56.7	59.8	43.5	68.6	34.5	3.6
*P*-value		0.8	0.7	0.2	0.8	0.3	0.2	0.003
**Ethnicity**								
European/NZer	60.3 (3403)	76.5	63.2	65.5	48.6	62.9	21.9	2.2
Māori	13.4 (759)	58.8	42.8	48.4	31.4	81.5	45.9	7.4
Pacific	13.1 (742)	58.8	47.7	49.7	38.5	80.5	57.2	13.1
Asian	11.8 (669)	57.6	51.8	47.8	39.0	75.8	57.6	2.7
MELAA	1.3 (72)	68.1	58.8	58.8	47.2	73.6	34.7	5.6
*P*-value		<0.001	<0.001	<0.001	<0.001	<0.001	<0.001	<0.001
**Household NZ Deprivation index (decile range)**								
Low (1–3)	28.4 (1576)	75.6	63.8	64.9	49.6	62.8	23.1	2.4
Medium (4–7)	37.5 (2082)	71.1	57.7	61.0	45.1	66.6	30.9	2.6
High (8–10)	34.1 (1893)	62.4	50.7	51.8	40.5	77.2	44.9	8.0
*P*-value		<0.001	<0.001	<0.001	<0.001	<0.001	<0.001	<0.001
**Maternal Education**								
No secondary school	6.1 (348)	51.1	38.1	37.7	27.0	83.9	40.1	7.2
Secondary school	22.5 (1277)	63.1	49.7	52.5	37.3	75.6	40.0	5.5
Diploma/trade	30.4 (1725)	67.2	53.8	56.9	43.1	74.4	37.0	6.3
Bachelor qualification	24.3 (1380)	73.9	62.9	65.0	49.0	63.7	26.6	1.9
Higher qualification	16.6 (944)	82.5	71.7	70.6	53.6	54.3	26.4	1.8
*P*-value		<0.001	<0.001	<0.001	<0.001	<0.001	<0.001	<0.001
**Food Security**								
Secure ≥26	49.6 (2564)	78.0	66.8	67.5	52.8	62.1	25.1	2.8
Insecure <26	50.4 (2605)	61.8	48.1	51.5	38.9	75.7	41.2	5.4
*P*-value		<0.001	<0.001	<0.001	<0.001	<0.001	<0.001	<0.001

**Table 2.** Prevalence of obesogenic behaviours (excessive screen time and short sleep duration) and obesity (WHO definition) in children exposed and not exposed to each of the three FSRs and exposed to different numbers of FSRs

Family screen rules	Prevalence of excessive screen time			Prevalence of short sleep duration			Prevalence of obesity		
	Has rule(s) % (95% CI)	Lack rule(s) % (95% CI)	Chi-square (*P*-value)	Has rule(s) % (95% CI)	Lack rule(s) % (95% CI)	Chi-square (*P*-value)	Has rule(s) % (95% CI)	Lack rule(s) % (95% CI)	Chi-square (*P*-value)
Rules on quality	66.8 (65.3–68.4)	79.5 (77.4–81.5)	81.6 (<0.001)	30.8 (29.3–32.4)	41.4 (39.0–43.9)	53.20 (<0.001)	4.0 (3.3–4.6)	6.1 (5.0–7.4)	11.59 (<0.001)
Rules on quantity	63.9 (62.1–65.6)	79.6 (77.8–81.2)	145.5 (<0.001)	29.9 (28.2–31.6)	39.4 (37.4–41.5)	49.9 (<0.001)	3.5 (2.9–4.3)	5.9 (5.0–7.0)	16.36 (<0.001)
Rules on timing	65.0 (63.3–66.7)	78.6 (76.7–80.3)	107.1 (<0.001)	30.3 (28.7–32.0)	39.1 (37.0–41.3)	41.66 (<0.001)	3.9 (3.2–4.6)	5.4 (4.6–6.5)	7.27 (0.007)
None (no rule)	73.2 (71.1–75.1)		90.1 (<0.001)	39.3 (37.1–41.5)		39.0 (<0.001)	5.3 (4.4–6.3)		11.67 (<0.009)
Only one rule	79.4 (75.5–82.8)			32.8 (28.7–37.2)			5.1 (3.4–7.4)		
Any two rules	73.6 (70.2–76.6)			31.5 (28.2–34.9)			5.3 (3.9–7.2)		
All three rules	62.8 (60.9–64.7)			30.0 (28.2–31.8)			3.4 (2.7–4.2)		

Prevalence of households with no FSR, only one FSR, any two FSRs, and all three FSRs in the whole sample were 2005 35.0% (*n* = 2005), 8.3% (*n* = 475), 13.1% (*n* = 749), and 43.7% (2504), respectively.

Logistic regression models were used to estimate the odds of obesity and obesogenic behaviours associated with exposure to each and different numbers of FSRs, presenting unadjusted odds ratios (ORs) and adjusted odds ratios (AORs) after accounting for covariates (Table 3).

**Table 3.** Association between family screen rules (FSRs), obesity (WHO definition), and obesogenic behaviours (excessive screen time and short sleep duration)

	Excessive screen time (≥1 h/day)		Short sleep duration (<10 h/night)		Obesity	
Family screen rules (FSRs)	UOR	AOR^a^	UOR	AOR^a^	UOR	AOR^a^
Rule on quality^b^	0.52 (0.45–0.60)	0.65 (0.56–0.76)	0.63 (0.55–0.71)	0.84 (0.73–0.97)	0.63 (0.48–0.82)	0.89 (0.65–1.20)
Rule on quantity^b^	0.45 (0.40–0.52)	0.53 (0.46–0.61)	0.65 (0.58–0.74)	0.81 (0.71–0.92)	0.58 (0.45–0.76)	0.79 (0.59–1.07)
Rule on timing^b^	0.51 (0.44–0.58)	0.61 (0.53–0.70)	0.68 (0.60–0.76)	0.87 (0.76–0.99)	0.70 (0.53–0.91)	0.97 (0.72–1.30)
None (no rule)	Ref	Ref	Ref	Ref	Ref	Ref
Only one rule	1.41 (1.10–1.81)	1.40 (1.08–1.82)	0.75 (0.61–0.93)	0.82 (0.64–1.04)	0.95 (0.60–1.50)	0.93 (0.54–1.58)
Any two rules	1.02 (0.84–1.24)	1.18 (0.96–1.45)	0.71 (0.59–0.85)	0.95 (0.78–1.16)	1.01 (0.69–1.47)	1.51 (1.00–2.30)
All three rules	0.62 (0.54–0.71)	0.72 (0.63–0.84)	0.66 (0.58–0.75)	0.84 (0.73–0.97)	0.63 (0.47–0.84)	0.83 (0.59–1.16)

a AORs are adjusted for child’s age, sex, ethnicity, area level deprivation index, maternal education, and food security.

b Reference group for all models related to individual FSRs was those with lack of a given rule.

In the second stage of analysis, SEM was used to develop and test a model linking FSRs to child zBMI. The results from the first stage, complemented by correlation analyses and existing literature regarding the association between FSRs, screen time, sleep duration, and child weight informed the inclusion/exclusion of variables in the subsequent analyses. In this modelling, the FSRs variable was examined as both a direct and an indirect predictor of childhood zBMI.

**Figure 1.**
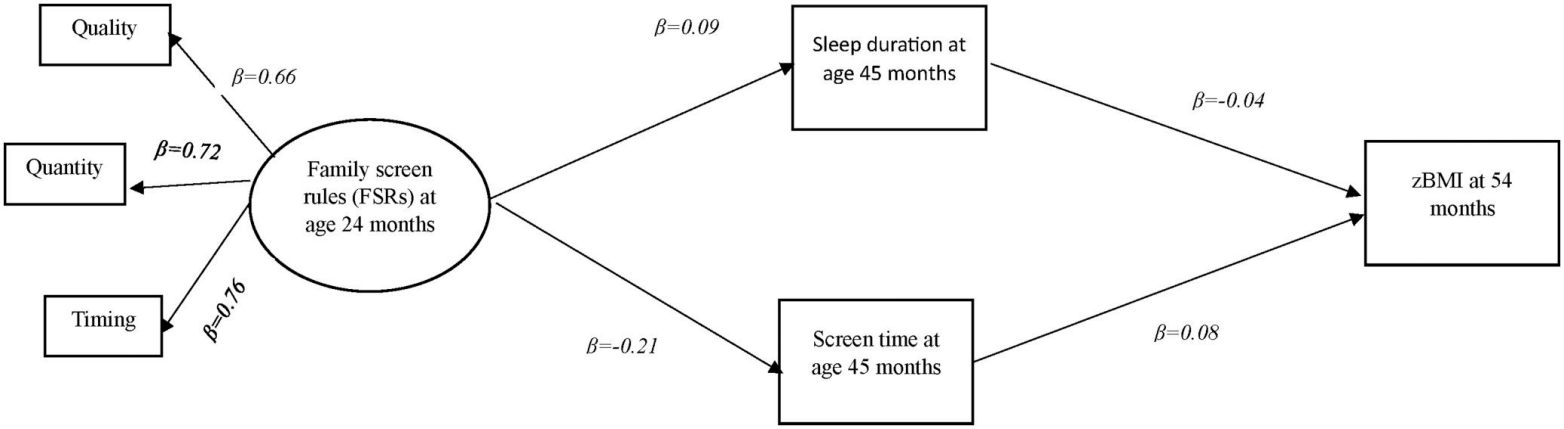
Final model linking family screen rules (at age 24 months) to childhood BMI (at age 54 months) through sleep duration and screen time (at age 45 months).

The three screen-based rules were reduced to one latent variable representing FSRs, without applying any differential weightings. Observed continuous variables were used to represent screen time, sleep duration, and child zBMI. The continuous food security variable was incorporated into the model as a covariate (not shown in Fig. 1). For simplicity, other covariates were excluded from this model as their adjustment did not alter the model fit indices. Variable distributions were assessed for normality and extreme values using skew and kurtosis indices and normal quantile plots. The analyses were conducted using complete case analysis, whereby missing data were deleted list-wise. This resulted in a final sample size of 3974 children. Maximum Likelihood was employed as the estimation method in all models.

Model fit for SEM was assessed using widely accepted relative fit indices, including the comparative fit index (CFI) ≥0.90, the goodness of fit (GFI) ≥0.95 or adjusted goodness of fit (AGFI) ≥0.90, the root mean square error of approximation (RMSEA) ≤0.08, standardized root mean square residual (SRMR) <0.08 [33, 34]. The significance level for the model *z*-test was set at *P* < 0.05. To address the multiple mediations present in our model (specifically, two mediating paths within a single model), we employed the bootstrap method [35]. Percentile bootstrap confidence intervals were reported to assess the significance of the indirect effects. Unstandardized path coefficients (b), standard errors, and *P*-values were reported alongside standardized path coefficients (β) to facilitate the interpretation of associations.

Descriptive analyses and logistic regression models were performed using Stata 15 [36]. For SEM, Amos software was utilized [37].

## Results

### Sample characteristics

At the fifth data collection wave, child ages ranged from 48.5 to 62.5 months (mean = 54.5, SD = 1.53). The prevalence of obesity was 4.5%. Girls comprised 48% of the sample, of whom nearly half (49.6%) were identified as food insecure at 9 months. About one-third lived in areas of high deprivation, and 28.6% had a mother with educational attainment at or below high school level.

At age 24 months, 43.8% of children were exposed to all three FSRs, 21.4% to one or two, and 35% were exposed to none. Over two-thirds (69.2%) of the children exceeded the recommended daily limit of screen viewing (<1 hour/day), and 33.6% were reported to sleep less than the recommended 10 h/night.

The prevalence of exposure to all three FSRs, excessive screen time (≥1 hour/day), and short sleep duration (<10 h/night) did not differ significantly by the child’s gender. However, obesity was more prevalent among boys compared with girls (5.23% vs. 3.62%, respectively). Obesity, short sleep duration, and excessive screen time were more prevalent among children identified as food insecure, residing in areas of higher deprivation, with maternal education at secondary or lower levels, and those identified as Māori or Pacific (Table 1).

### Association between FSRs and obesity

After adjusting for sociodemographic characteristics (including child’s gender, age, and self-prioritized ethnicity, maternal prenatal education level, food security, and neighbourhood deprivation level), none of FSRs at age 24 months were significantly associated with lower odds of obesity at age 54 months at the individual level. Likewise, exposure to a greater number of FSRs was not associated with obesity after adjusting for sociodemographic characteristics at the cumulative level (Table 3). These results were consistent with those obtained using SEM, where the direct association between FSRs and child zBMI was not significant.

### Association between FSRs and screen time

At the individual level, after adjusting for covariates, odds of exceeding the 45-month screen viewing guideline (≥1 hour/day) were lower among children exposed to any given FSR compared to children not exposed to that rule at age 24 months; for the rule on quality AOR = 0.65, 0.56–0.76; the rule on quantity AOR = 0.53, 0.46–0.61; the rule on timing AOR: 0.61, 0.53–0.70 (Table 3).

At the cumulative level, after adjusting for covariates, only having all three FSRs was significantly associated with decreased odds of exceeding the 45-month screen viewing guideline (AOR = 0.73, 0.63–0.84). Similarly, using SEM, a negative association was found between exposure to FSRs (latent variable) and child’s screen time at age 45 months (continuous variable).

The odds of exceeding the 45-month screen viewing guideline were higher for children exposed only to one FSR compared to those with no exposure (AOR = 1.40, 1.08–1.82). However, exposure to two FSRs was not significantly associated with exceeding the 45-month screen viewing guideline (Table 3).

### Association between FSRs and sleep duration

At the individual level, after adjusting for covariates, odds of sleeping less than the recommended 10 h/night were lower among children exposed to any given FSR compared to children not exposed to that rule; for the rule on quality AOR = 0.84, 0.73–0.97; the rule on quantity AOR = 0.81, 0.71–0.92; the rule on timing AOR: 0.87, 0.76–0.99. At the cumulative level, after adjusting for sociodemographic characteristics, only having all three FSRs was significantly associated with lower odds of sleeping less than the recommended time (AOR = 0.84, 0.73–0.97) (Table 3). Similarly, using SEM, a positive association was found between exposure to FSRs and child’s sleep duration at age 45 months.

### The mediating role of obesogenic behaviours in the association between FSRs and zBMI

An initial model assessed both direct and indirect paths between FSRs and child zBMI via screen time and sleep duration. However, the direct path was non-significant (*P* = 0.08) and removed from the final model.

The final model depicts only the indirect effect of FSRs on zBMI through sleep duration and screen time. All path coefficients were standardized to facilitate interpretation of effect magnitudes. Paths linking the covariate variable (food security) to other variables were omitted for clarity (Fig. 1).

In the final model, FSRs were positively associated with sleep duration (β = 0.09, *P* < 0.001) and negatively associated with screen time (β = −0.21, *P* < 0.001). Sleep duration was negatively (β = −0.04, *P* = 0.01) and screen time was positively (β = 0.08, *P* < 0.001) associated with zBMI at age 54 months. The indirect (mediated) effect of FSRs on zBMI was significant (β= −03, *P* = 0.004, two-tailed, bootstrapped 95% CI: −0.003, −0.13). The final model indicated good fit with the data (CFI = 0.97, GFI = 0.99, AGFI = 0.97, RMSEA = 0.05, SRMR = 0.03).

## Discussion

This study examined the association between three FSRs (rules on screen quality, quantity, and timing) at 24 months, obesogenic behaviours at 45 months, and child BMI at 54 months, as well as the potential mediating role of these behaviours in the FSR–obesity link.

Our analysis of a large, nationally representative sample of NZ preschoolers revealed that only 43.7% of children were exposed to all three FSRs by age 24 months, with lower prevalence among Māori, Pacific, Asian, and economically disadvantaged children. By 45 months, children averaged nearly 3 hours of daily screen time, well above the recommended 1-hour limit. However, no significant association was found between FSRs at 24 months and obesity at 54 months, after adjusting for sociodemographic factors. SEM findings also revealed no direct link between FSRs and zBMI.

This aligns with previous research [12, 14] indicating that FSRs regulating screen viewing time did not directly reduce obesity risk. However, findings contrast with those reported by Johnson et al., which linked the absence of FSRs to increased zBMI [13].

The study’s second objective investigated whether FSRs influenced a child’s obesogenic behaviours. Findings showed that children exposed to individual FSRs or all three FSRs at 24 months had lower odds of not meeting screen time and sleep guidelines at 45 months, reinforcing existing evidence linking TV rules to reduced TV viewing [12, 14].

The protective effects of FSRs for sleep duration could be due to a negative association between screen time and sleep duration found in previous research [17], suggesting that children who spend less time on screen devices are more likely to get adequate sleep. It is also plausible that families with enforced rules around screen time may also implement other rules regulating sleep behaviours, leading to improved sleep outcomes for children.

Exposure to fewer than three rules did not significantly mitigate excessive screen time and short sleep duration at 45 months, suggesting multiple rules must be enforced to achieve a mitigating influence on obesogenic behaviour. These findings underscore the importance of consistent messaging across various aspects of FSRs.

Next, we examined whether FSRs influence zBMI indirectly through the mediating role of screen time and sleep duration using a SEM approach. While no direct path was found between FSRs and zBMI, a significant indirect association emerged through screen and sleep behaviours.

Notably, FSRs were negatively associated with screen time, positively associated with sleep duration, and both behaviours influenced zBMI. These findings align with prior research suggesting that FSRs play a protective role against excessive screen time [11, 13], itself a factor associated with lower zBMI [12, 38]. Additionally, our longitudinal analysis revealed that increased exposure to FSRs at 24 months corresponded with extended sleep duration by age 45 months, ultimately contributing to lower zBMI by age 54 months.

The shift from significant unadjusted associations to non-significant results after adjustment for socioeconomic factors suggests that these factors play a crucial role in the link between FSRs and childhood obesity. This indicates that the impact of FSRs on obesity may be heavily influenced by underlying sociodemographic variables, which may be stronger determinants of obesity than FSRs alone. To be effective, FSRs should be part of broader strategies that address these socioeconomic factors, especially in financially disadvantaged families who may need additional support to implement and maintain FSRs.

Align with ecological model, these findings emphasize the multiple levels of influence on a child’s outcome, recognizing that childhood obesity is influenced by a complex interplay of various factors such as family environment and socioeconomic status as found in previous research [39]. The results hold significant implications for the design of family-based initiatives to prevent and address childhood obesity and suggest that interventions focused on regulating screen time across various household domains could effectively reduce children’s screen time and improve sleep duration, ultimately contributing to a lower risk of developing childhood obesity. Active involvement of parents in such initiatives is crucial, as they play a pivotal role in regulating these behaviours in young children. Furthermore, the enduring effects of implementing FSRs at age two on child health behaviours and BMI in middle childhood highlight the importance of early intervention.

Study strengths include its utilization of a robust longitudinal design and a nationally representative sample, enhancing the reliability and generalizability of findings. Furthermore, examining three specific FSRs and their longitudinal impacts on childhood obesity—both directly and indirectly—provides a nuanced understanding of the complex relationship between FSRs and children’s health outcomes. The study’s incorporation of rule enforcement in distinguishing families with and without rules also strengthens its validity, allowing for more precise estimates of the effectiveness of such rules [14] and potential impact on behaviour change. Further, the use of multiple specific rules adds depth compared to previous studies focusing on single rules or broader categories without delineating their specific nature [12, 13]. Understanding each rule’s characteristics helps tailor interventions more effectively to target specific areas of concern. Additionally, the study broadens the scope beyond television viewing to include other screen devices, crucial for addressing sedentary behaviours comprehensively [4, 14].

The study has limitations. Firstly, its observational design and correlational analyses prevent establishing a definitive causal link between FSR adoption and reduced obesity risk. Although the longitudinal approach and dose-response associations suggest a potential causal relationship, intervention studies are needed to confirm FSR effectiveness in improving health behaviours in combating obesity. Additionally, key confounding factors like children’s physical activity levels, programme content, advertising exposure, co-viewing with parents, and dietary patterns were not accounted for due to data unavailability, potentially influencing the results as suggested in previous research [40]. Future studies should incorporate these variables, along with other relevant factors like daycare attendance, to gain a more comprehensive understanding of the relationship between FSRs and childhood obesity. Moreover, investigating how FSRs impact overweight could provide further insights into their broader effects on weight outcomes. Lastly, data on FSRs relied on maternal reports, susceptible to recall or interpretation biases.

In conclusion, this research has shed light on the relationship of FSRs with child obesity, exploring both direct and indirect pathways. Our findings underscore the importance of promoting the adoption of FSRs as a promising population-based approach to improve child health behaviours, thereby reducing the risk of childhood obesity. Healthcare providers, educators, and other professionals working with families to address childhood obesity are encouraged to advocate for the implementation of family rules as a valuable strategy in their interactions with parents.

## Supplementary Material

ckae169_Supplementary_Data

## Data Availability

Data used in this research can be available through request from the GUINZ Data Access Committee (reachable at: Dataaccess@growingup.co.nz). Data access will be subject to the conditions set out in Growing Up in New Zealand Data Access Protocol and subject to review by the Data Access Committee. Key points The study explored FSRs’ influence on childhood obesity through obesogenic behaviours. FSR exposure correlated with reduced odds of excessive screen time and inadequate sleep, suggesting protective effects. While direct FSR–obesity associations were non-significant, indirect pathways via obesogenic behaviours were significant. These findings emphasize the potential of FSR promotion as a strategy to mitigate childhood obesity risk. Tailored interventions and additional support may be needed for financially disadvantaged families to implement FSRs effectively.
