# Correction to: Prospective relationship between family screen time rules, obesogenic behaviours, and childhood obesity

**DOI:** 10.1093/eurpub/ckae207

**Published:** 2024-12-15

This is a correction to: Ladan Hashemi, Maryam Ghasemi, Deborah Schlichting, Maryam Pirouzi, Cameron Grant, Boyd Swinburn, Prospective relationship between family screen time rules, obesogenic behaviours, and childhood obesity, European Journal of Public Health, 2024, ckae169, https://doi.org/10.1093/eurpub/ckae169

In the originally published version of this manuscript, subheadings were inadvertently omitted from Table 1.

This error has been corrected.

