# Supplementary figures and images for: Prospective relationship between family screen time rules, obesogenic behaviours, and childhood obesity
Source: Eur J Public Health. 2024 Nov 18;35(1):114–20. doi: 10.1093/eurpub/ckae169 (PMC11832151; doi:10.1093/eurpub/ckae169)

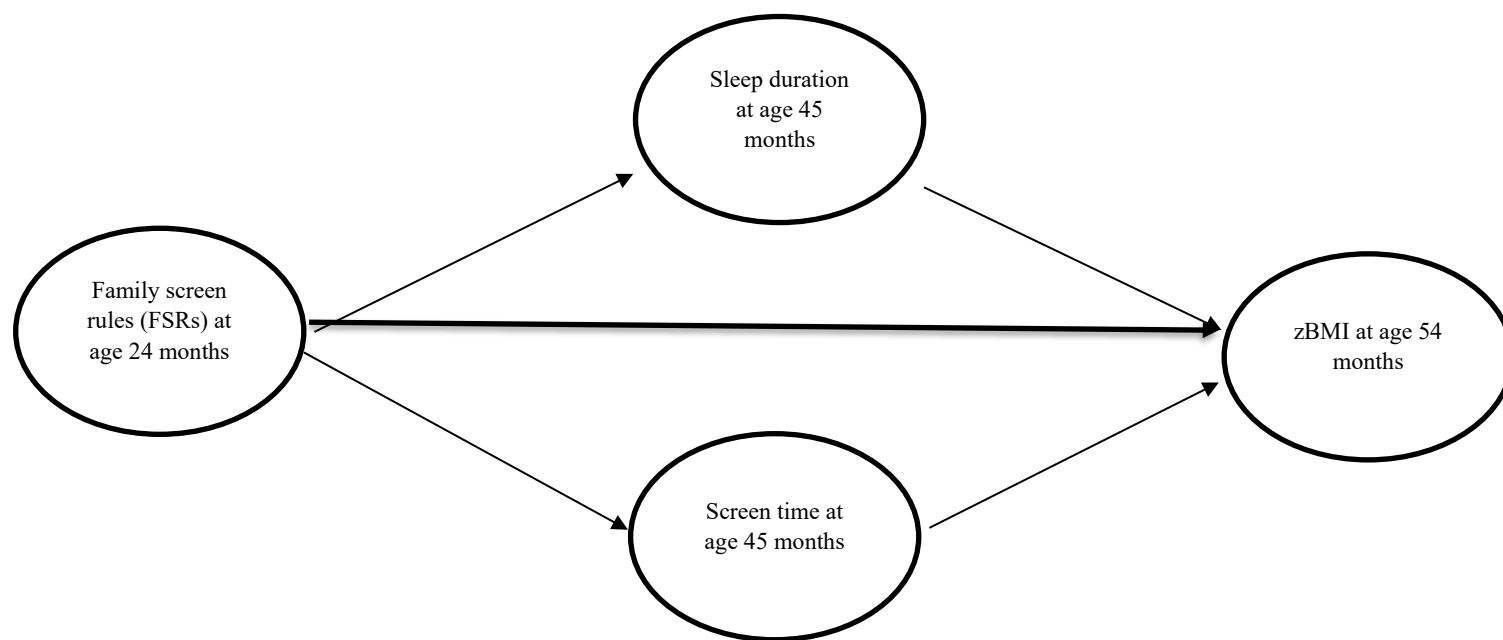

**Supplementary Figure 1. The proposed model**

Supplement: ckae169_Supplementary_Data [file ckae169_supplementary_data.pdf]
